# Highly Sensitive and Stretchable c-MWCNTs/PPy Embedded Multidirectional Strain Sensor Based on Double Elastic Fabric for Human Motion Detection

**DOI:** 10.3390/nano11092333

**Published:** 2021-09-08

**Authors:** Huiying Shen, Huizhen Ke, Jingdong Feng, Chenyu Jiang, Qufu Wei, Qingqing Wang

**Affiliations:** 1 Key Laboratory of Eco-Textiles, Ministry of Education, Jiangnan University, Wuxi 214122, China; shenhuiying_vicky@163.com (H.S.); 13101972828@163.com (J.F.); qfwei@jiangnan.edu.cn (Q.W.); 2 Key Laboratory of Novel Functional Textile Fibers and Materials, Minjiang University, Fuzhou 350108, China; kehuizhen2013@163.com; 3 Department of Chemistry, North Carolina State University, Raleigh, NC 27695, USA; cjiang13@ncsu.edu

**Keywords:** double elastic fabric, carbon nanotube, polypyrrole, multidirectional, strain sensor, complicated human motions detection

## Abstract

Owing to the multi-dimensional complexity of human motions, traditional uniaxial strain sensors lack the accuracy in monitoring dynamic body motions working in different directions, thus multidirectional strain sensors with excellent electromechanical performance are urgently in need. Towards this goal, in this work, a stretchable biaxial strain sensor based on double elastic fabric (DEF) was developed by incorporating carboxylic multi-walled carbon nanotubes(c-MWCNTs) and polypyrrole (PPy) into fabric through simple, scalable soaking and adsorption-oxidizing methods. The fabricated DEF/c-MWCNTs/PPy strain sensor exhibited outstanding anisotropic strain sensing performance, including relatively high sensitivity with the maximum gauge factor (GF) of 5.2, good stretchability of over 80%, fast response time < 100 ms, favorable electromechanical stability, and durability for over 800 stretching–releasing cycles. Moreover, applications of DEF/c-MWCNTs/PPy strain sensor for wearable devices were also reported, which were used for detecting human subtle motions and dynamic large-scale motions. The unconventional applications of DEF/c-MWCNTs/PPy strain sensor were also demonstrated by monitoring complex multi-degrees-of-freedom synovial joint motions of human body, such as neck and shoulder movements, suggesting that such materials showed a great potential to be applied in wearable electronics and personal healthcare monitoring.

## 1. Introduction

In recent years, flexible strain sensors have experienced rapid development and achieved a series of progresses in many fields like soft robotics [1], artificial skin [2], human motion detection [3,4], personal health monitoring [5], and human-machine interface [6]. To get better sensitivity and larger workable range, which are the main parameters of the strain sensor, many conductive materials based on semiconductors, nanomaterials, and conductive polymers were used to design strain sensitive sensors, such as ZnO [7,8], ZnSnO_3_ [9], silver nanowire [10], silver nanoparticles [11], carbon nanotube [12,13], graphene [14,15,16], polypyrrole [17], and polyaniline [18], have been applied and coupled to stretchable substrates. These successfully prepared sensors can respond quickly to a large range of testing strain with high stretchability and sensitivity. Examples of such flexible sensors include carbonized silk fabric and cotton fabric [19], conductive film made of graphene and ionic conductor [20], thin slice composed of silver nanoparticles and nanowires [21], and conductive fabrics that are polypyrrole-coated [17].

Although these reported strain sensors possessed good extensibility and sensitivity (i.e., high gauge factor (GFs) value and strain range), they lacked the capability to monitor motions working in different directions, as they can only detect strain in a single-direction such as finger and knee joints motions (i.e., single axial joints motions), which limited their wider applications in detecting more complicated multiaxial, multi-dimensional strain conditions and interfacing with multi-degrees-of-freedom machines, etc. [22,23]. Multidimensional sensors with distinguishable signals for simultaneous detection of complex postures and movements in multiple directions are in high demand for the development of wearable electronics [24]. To overcome the critical limitation of the conventional unidirectional sensors, several attempts have been made to establish wearable devices with anisotropic electromechanical behaviors [25]. For example, a previous work presented a multidirectional strain sensor composed of a monolithic integration of a stiffness-variant stretchable substrate and a sensing film to detect the multidimensional strains of the human motions, which could distinguish strains in various directions [26]. A prestrained silver nanowire percolation network film was designed using vacuum filtration and transfer method to fabricate a biaxial strain sensor that can detect the resistance change of the x and y axes independently [27]. Very recently, a skin-inspired multidimensional sensor capable of sensing 3D stimuli is developed by integrating various selective sub-sensors with 3D anisotropic structure, which can simultaneously and selectively measure strains in three orthogonal axes. This multidimensional sensor is used in a smart sport assistant device, which can monitor sport performance and provide feedback in real-time [28]. Moreover, multidimensional sensors using three-dimensional (3D) printing techniques were developed and integrated with soft robotic actuators for detecting the multidirectional rolling detection and distinguishing bending movement [29].

Another strategy to fabricate anisotropic strain sensors is to utilize conductive materials embedded textile materials, which consist of yarn or fibers directly. Textile fabric possesses some intriguing features such as low cost, light weight, excellent flexibility, high strength, and outstanding recovery properties [30,31,32], demonstrating their promise as a substrate for strain sensor applications. Previous studies have shown that a flexible fabric-based strain sensor mounted on the human skin or integrated into the clothing can exhibit a real-time and stable response to monitor human activities, and can accurately distinguish diverse human motions [33,34,35]. However, few fabric-based sensors are capable of detecting the resistance change of the x and y axes, which set higher demands for sensor substrate. One such scaffold is double elastic fabric (DEF), which could be stretched along both of warp and weft direction, can be used for monitoring complex multidirectional human motions after conductive treatment. To the best of our knowledge, fabrication methods that combine fabric with conductive sensing elements to develop flexible fabric-based strain sensors including carbonization [36], depositing [37,38,39], or coating [40] method to form conductive networks.

Given the flexibility of double elastic fabric as a substrate, and the use of c-MWCNTs and PPy with relatively low cost, excellent mechanical, electrical, and thermal conductivity properties to function as conductive agents, herein we developed a stretchable biaxial strain sensor (termed DEF/c-MWCNTs/PPy) by dip-coating in c-MWCNT dispersions and adsorption-oxidizing pyrrole methods to realize real-time monitoring of complicated human body motions. The morphology (SEM), elemental composition (EDS and XPS), and the electromechanical properties of the obtained materials were systematically characterized. A series of human body motions including subtle motions and dynamic large-scale motions were performed to demonstrate the high sensitivity, good stretchability, highly responsive, outstanding stability, and durability of DEF/c-MWCNTs/PPy strain sensors. Furthermore, the complex multi-degrees-of-freedom synovial joint motions of human body were also detected to achieve multidirectional monitoring. Taken together, our findings will manifest that the DEF/c-MWCNTs/PPy strain sensor has favorable anisotropic sensing property, and thus demonstrate this functional material as a promising candidate for use in wearable electronics and personal healthcare monitoring.

## 2. Materials and Methods

### 2.1. Materials

Commercially available polyester/cotton spandex core-spun yarn (JCVC 40 S/2/A70D) and cotton spandex core-spun yarn (C40 S/2/A70D) with twill weave structure (2/1, 288 × 201/10 cm) woven by Danyang Dansheng Textile Co., Ltd. (Changzhou, China) were used as raw materials. Sodium hydroxide (NaOH), bovine serum albumin (BSA), isopropyl alcohol, and ferric chloride (FeCl_3_) were purchased from Sinopharm Chemical Reagent Co., Ltd. (Shanghai, China). Carboxylic Multiwalled carbon nanotubes (c-MWCNTs, outer diameter 5–15 nm, length 10–30 μm and carboxyl content 3.86 wt.%) were obtained from Nanjing XFNANO Materials Tech Co., Ltd. (Nanjing, China), while pyrrole was supplied by Aladdin Reagent Co., Ltd. (Shanghai, China). Deionized water was used for all experiments, and all chemicals were used as received.

### 2.2. Preparation of DEF/c-MWCNTs/PPy Strain Sensors

Initially, the pristine double elastic fabric (DEF) needed to be pretreated to remove impurities from the fabric surface. Briefly, the fabric was firstly placed into a beaker containing 1 M NaOH aqueous solution and soaked in a water bath at 80 °C (liquor ratio 1:50) for 30 min with continuously stirring for desizing treatment. Subsequently, the desized fabric was sonicated in deionized water for 10 min to remove impurities and then dried at 50 °C in a drying oven (DHG-9030A, Shanghai Yiheng Scientific Instrument Co., Ltd., Shanghai, China). Subsequently, the dried fabric was immersed into 0.5 wt.% BSA aqueous solution for 30 min in a 37 °C orbital shaker (CHA-SA, Changzhou Putian Instrument Manufacturing Co., Ltd., Changzhou, China) at a speed of 80–100 rpm. Finally, the BSA-decorated DEF was obtained after being washed by deionized water for three times.

The c-MWCNT-loaded fabric was developed by dipping method. Firstly, c-MWCNT powder was dissolved in deionized water and sonicated for 2 h to prepare 0.2 wt.% homogeneous c-MWCNT solution. The BSA-decorated DEF was then soaked into the c-MWCNT solutions (200 mL) overnight to fully adsorb the c-MWCNT. Subsequently, the fabric was taken out and rinsed with deionized water for several times to wash off any unbound c-MWCNT. Notably, the dipping procedure was repeated five times. For further fabricating, the PPy and c-MWCNT immobilized fabric, adsorption-oxidizing method was employed. Firstly, the prepared DEF/c-MWCNT was immersed into a 1 M FeCl_3_ solution for 10 s, then quickly removed and placed in a 0.5 M pyrrole isopropanol solution for 10 s. Afterward, the fabric was put in a fume hood for 10 min to thoroughly react, and was followed by repeatedly washing with DI water and drying. The adsorption-oxidizing process was repeated six times until the desired DEF/c-MWCNT/PPy was obtained.

The acquired DEF/c-MWCNTs/PPy were tailored to an appropriate rectangle for further use (6 cm × 2 cm, 3 cm × 1 cm, 2 cm × 0.5 cm, 2 cm × 2 cm). Both ends of the DEF/c-MWCNTs/PPy were connected with copper wires and copper foils, then were encapsulated with PU membrane waterproof tape to prepare DEF/c-MWCNTs/PPy strain sensor.

### 2.3. Characterization

To observe the morphology of DEF before and after c-MWCNTs and PPy coating, scanning electron microscopy (SEM, Hitachi SU1510, Tokyo, Japan) was conducted at an accelerating voltage of 5 kV with a magnification of 3 K. To verify the successful loading of c-MWCNTs and PPy on the fabric and analyze the elemental composition of the obtained material, Energy-dispersive spectroscopy (EDS) mapping was performed on an EDAX Octane Elect ESD-30 System (EDAX Inc., Philadelphia, PA, USA with a working voltage of 15 kV, while X-ray photoelectron spectroscopy (XPS) was accomplished using a Thermo Scientific K-Alpha (Waltham, MA, USA) spectrometer.

The electronic and mechanical performance of DEF/c-MWCNTs/PPy strain sensor was recorded with the PT-1198GDT uniaxial testing machine (Perfect International Instruments Co., Ltd., Dongguan, China) at a deformation rate of 50 mm/min and a push–pull force gauge (Handpi Co., Ltd., Leqing, China) with a clamp distance of 2 cm. The current-voltage (I-V) curve was collected on an electrochemical workstation (CHI 660E, CH Instruments Inc., Austin, TX, USA). The resistance signals under stretching-releasing test were measured with a Keithley 2450 source meter and a 6.5 Digital Multimeter (34401A, Agilent Technologies Co., Ltd., Palo Alto, CA, USA). The tensile test of pristine double elastic fabric was carried out by the YG 026T electronic tensile testing machine (Wenzhou Darong Textile Instrument Co., Ltd., Wenzhou, China), with the size of 50 × 200 mm, and the clamping distance, pre-tension and the tensile speed were set as 100 mm, 2 N, and 100 mm/min, respectively.

All the experiments involving the human volunteers were performed with full compliance with all local laws and institutional ethical guidelines. Full and informed consent was given by each subject for these experiments.

## 3. Results and Discussion

### 3.1. Fabrication and Characterization of DEF/c-MWCNTs/PPy Strain Sensor

**Figure 1:**
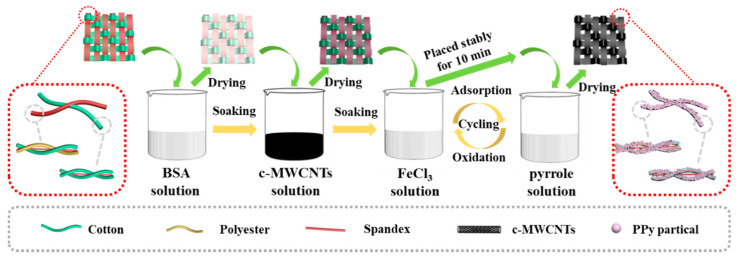
Schematic illustrations of fabrication procedure of DEF/c-MWCNTs/PPy strain sensor.

The process of preparing the DEF/c-MWCNTs/PPy strain sensor is shown in Figure 1. The double elastic fabric used cotton polyester/spandex core-spun yarn as the warp yarn and cotton/spandex core-spun yarn as the weft yarn, which exhibited gratifying strain when stress was applied along both the weft and warp direction. The DEF/c-MWCNTs/PPy strain sensor was fabricated by soaking and adsorption-oxidizing methods. As reported previously [41], the DEF was initially immersed into positively charged bovine serum albumin (BSA) solution, and then soaked in c-MWCNTs solution. As the amphoteric substances [42], the BAS could be adsorbed onto the fabric surface due to the existence of a large number of hydroxyl groups of cotton [43]. Moreover, the positively charged BSA could combine the negatively charged carboxylic multi-walled carbon nanotubes (c-MWCNTs) more stably through electrostatic adherence. Subsequently, the DEF/c-MWCNTs/PPy strain sensor was obtained after six cycles of adsorption-oxidizing in FeCl_3_ and pyrrole solution, respectively.

**Figure 2:**
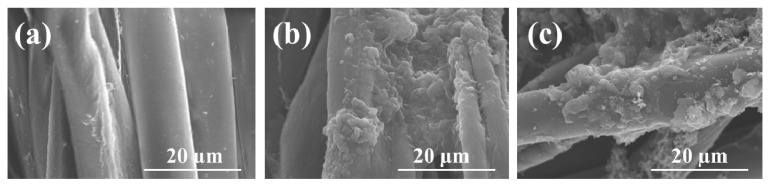
SEM images of (**a**) pristine cotton fibers and polyester fibers. (**b**) Diber-loaded c-MWCNTs and (**c**) fiber-loaded c-MWCNTs and PPy.

**Figure 3:**
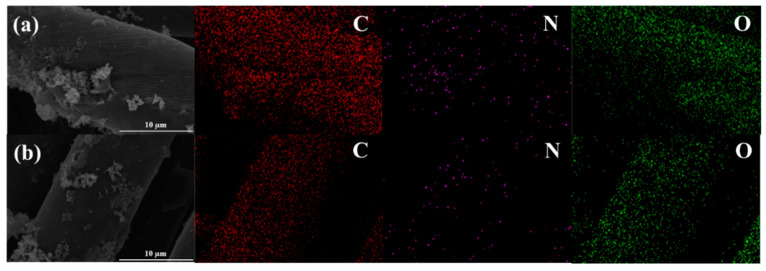
EDS mapping of double elastic fabric-loaded c-MWCNTs and PPy: (**a**) cotton fibers; (**b**) polyester fibers.

The typical surface morphologies and elemental distribution of individual polyester fiber and cotton fiber in the lengthwise warp core spun yarn before and after c-MWCNT-PPy immobilization were investigated by SEM and EDS elemental mapping. As a synthetic fiber, polyester fiber was smooth in the longitudinal direction, while cotton fiber showed a characteristic twisted ribbon-like structure with some convolutions in longitudinal direction, which endowed the cotton a relatively uneven surface (Figure 2). By contrast, after the c-MWCNT deposition, it was observed that the c-MWCNT formed a uniform coating on the fiber surface, which led to a compact structure with the fibers closely adhered to each other. The integration of PPy to the c-MWCNT-immobilized fabric resulted in a massive agglomeration on the c-MWCNT-layer with some blocks-shaped PPy appearing on the fiber surface or embedded in the space between fibers. Furthermore, as shown in Figure S1, an even intertwined web of c-MWCNT and PPy could be observed in lager magnification. EDS elemental mapping images (Figure 3) showed elements C, O, and N (characteristic elements of PPy) were uniformly distributed throughout the cotton fiber and polyester fiber, further confirming the successful loading of c-MWCNTs and PPy on the fabric surface.

**Figure 4:**
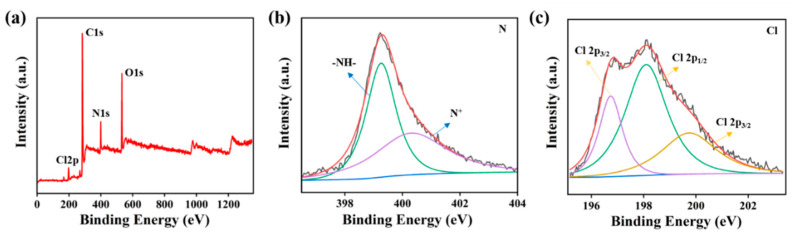
(**a**) XPS survey spectra, (**b**) N 1 s XPS spectra, (**c**) Cl 2 p XPS spectra of DEF/c-MWCNTs/PPy.

The obtained materials were further characterized via XPS to investigate the element composition and chemical structure (Figure 4). The XPS survey spectra (Figure 4a) confirmed the existence of C, N, O, Cl elements of DEF/c-MWCNTs/PPy. The N 1s spectra (Figure 4b) can be deconvolved into two prominent peaks at 399.3 and 400.4 eV, which were attributed to pyrrolylium nitrogen (-NH- structure) [44] and positively charged nitrogen (N^+^) [45], respectively. Figure 4c exhibited three spin-orbit split doublets (Cl 2p _3/2_ and Cl 2p _1/2_) of Cl 2p. The binding energy for Cl 2p _3/2_ located at about 196.7 and 199.7 eV were ascribed to the ionic and covalent chlorine species, respectively, and the binding energy for Cl 2p _1/2_ lying at 198.1 eV was associated with the species resulting from the charge transfer interaction between the chlorine and the conducting state of the PPy chain [44]. Taken together, these findings further demonstrated the successful loading of c-MWCNTs and PPy onto double elastic fabric.

### 3.2. Electromechanical Performances of DEF/c-MWCNTs/PPy Strain Sensor

**Figure 5:**
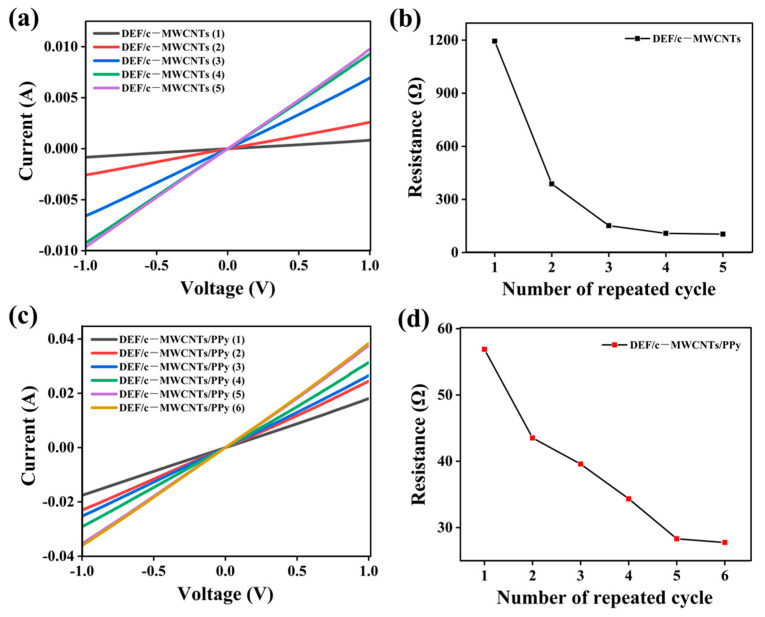
(**a**) The I–V plots of DEF/c-MWCNTs with different c-MWCNTs soaking cycles; (**b**) The relationship between resistance and the number of c-MWCNTs soaking cycles. (**c**) The I–V plots of DEF/c-MWCNTs/PPy with different of adsorption-oxidizing cycles of the PPy precursor; (**d**) The relationship between resistance and the number of adsorption-oxidizing cycles.

Figure 5 presented the current–voltage (I–V) curves of the DEF/c-MWCNTs/PPy strain sensor after different soaking times and adsorption-oxidizing cycles, and corresponding resistance. It can be seen that the sensor displayed an excellent linear I-V behavior and the slopes of the I–V curves progressively increased accompanied by the increased repeated cycles, corresponding to a continuously decreased resistance of the DEF/c-MWCNTs/PPy strain sensor (Figure 5a,c). After five times c-MWCNT soaking and six adsorption-oxidizing cycles, the resistance value reached the ideal demand, which was stable at 103 and 28 Ω, respectively (Figure 5b,d). Therefore, the DEF/c-MWCNTs/PPy strain sensor, after five times c-MWCNT soaking and six adsorption-oxidizing cycles, was chosen for further characterization.

**Figure 6:**
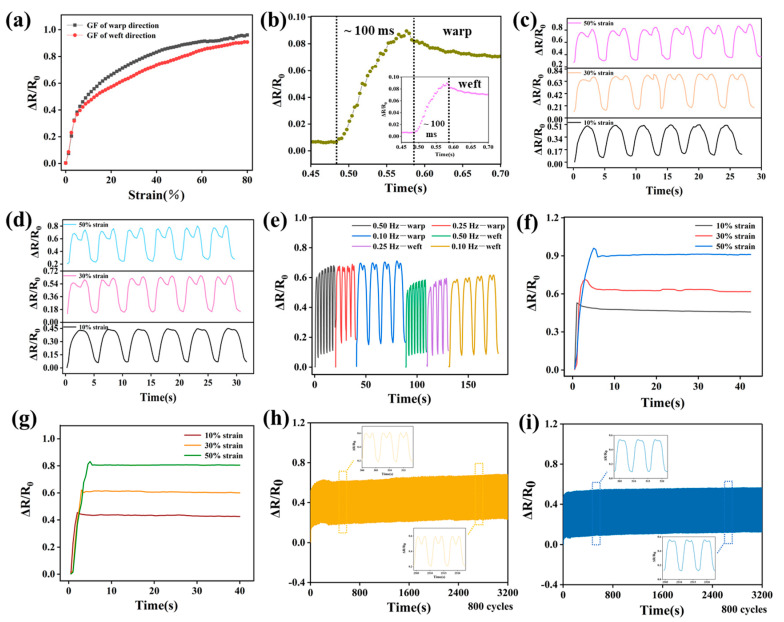
Strain sensing performance of the DEF/c-MWCNTs/PPy sensor. (**a**) Plots of RRV versus strain for DEF/c-MWCNTs/PPy sensor. (**b**) Response time of the DEF/c-MWCNTs/PPy sensor at a quasi-transient step strain of 1%. The RRV of the DEF/c-MWCNTs/PPy sensor under strains (10, 30, and 50%) at the frequency of 0.20 Hz: (**c**) warp sensor and (**d**) weft sensor. (**e**) The RRV of the warp and weft DEF/c-MWCNTs/PPy sensor at different frequencies (0.50, 0.25, and 0.10 Hz) under 20% strain. The RRV of the DEF/c-MWCNTs/PPy sensor for a step strain from 0 to 10%, 30 and 50% (strain rate 10% s-1): (**f**) warp sensor and (**g**) weft sensor. The stretching–releasing tests under a cyclic strain of 20% at 0.25 Hz for 800 cycles of (**h**) warp sensor and (**i**) weft sensor.

In order to further evaluate the electromechanical performance of DEF/c-MWCNTs/PPy sensor, gauge factor (GF), value was calculated by the slope of relative resistance variation (RRV) versus tensile strain. As shown in Figure 6a, the curve could be divided into two phases, initially, the resistance variation quickly increased in the strain range of 0–10% with the GF of warp sensor reaching 5.2 and weft sensor reaching 4.6, indicating an excellent resistance response of DEF/c-MWCNTs/PPy when applying strain. However, in the strain of 10–80%, a relatively slow increase for RRV was observed, the GF of warp sensor and weft sensor were 0.64 and 0.63, respectively. Compared to DEF/PPy sensor alone (Figure S2), whose maximum GF achieved 3.8 at the maximum strain of 20%, the addition of c-MWCNTs significantly enlarged the GF and strain range of DEF/c-MWCNTs/PPy sensor, fully demonstrating the superiority of the synergistic effect of c-MWCNTs and PPy on strain sensing. Additionally, the tensile strength tests of pristine DEF were presented in Figure S3, it was observed that the elongation at break of DEF was 80%, consistent with the strain range of the DEF/c-MWCNTs/PPy sensor. The comparison of the key performance indicators for different dual axis strain sensors was shown in Table S1.

Response time determines how quickly the strain sensors move toward steady state response. Figure 6b showed the response time of the DEF/c-MWCNTs/PPy sensor at a quasi-transient step strain of 1%, which was determined to be less than 100 ms, revealing that the sensor can make real-time monitoring and response to rapid and complex human activities.

Figure 6c,d illustrated the relative resistance change of DEF/c-MWCNTs/PPy warp direction and weft direction during repeated stretching/releasing cycles with 10, 30, and 50% strain at the frequency of 0.20 Hz. It was found that the curves of different stretching-releasing cycles were almost identical under a specific strain, manifesting the stability and repeatability of the sensor. Besides, for DEF/c-MWCNTs/PPy the intensity of RRV became higher gradually upon stretching to 10, 30, and 50%, which was capable of distinguishing different strain by forming different resistance signals.

The RRV of DEF/c-MWCNTs/PPy strain sensor during stretching/releasing process under same strain (20%), but with different frequencies (0.50, 0.25, and 0.10 Hz) was also investigated. As the Figure 6e depicted, the resistance signals was similar and the response was consistent with the change of frequencies. The signals could maintain good stability under specific frequencies, further confirming the repeatability of the strain sensor.

Moreover, the strain sensors exhibited overshoots with a short creep recovery time of 2.5, 3.5, and 6 s (warp sensor), along with 2.5, 3.5, and 5.5 s (weft sensor) when being stretched up to 10, 30, and 50% at a strain rate of 10% s^−1^ (Figure 6f,g), indicating the low creep of the strain sensors. After the recovery of the overshoot, the resistance signals of both the warp and weft sensor remained stable, suggesting the reliable performance of the strain sensors. Generally, the overshoot values are related to the GFs and strain rate of the strain sensors [46].

In addition, as displayed in Figure 6h,i, the durability of DEF/c-MWCNTs/PPy for both warp and weft direction were assessed through repeated stretching/releasing tests under a strain of 20%, and a frequency of 0.25 Hz was recorded for 800 cycles in real-time. It can be seen that the resistance signal remained relatively stable, the results indicated that both the warp and weft sensor possessed preferable electromechanical stability and durability, and had a long working life as strain sensors. Taken together, we found that DEF/c-MWCNTs/PPy strain sensors possessed relatively high sensitivity, fast response, low creep, and excellent durability.

### 3.3. Sensing Mechanisms of DEF/c-MWCNTs/PPy Strain Sensor

**Figure 7:**
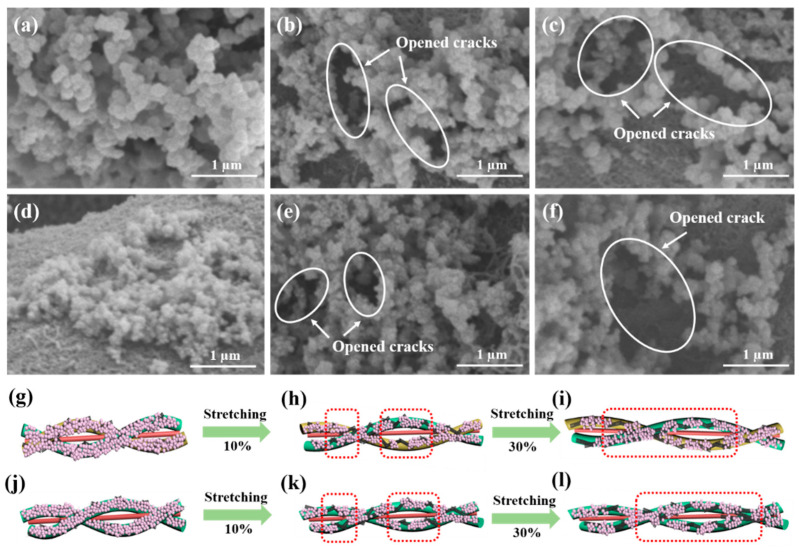
The sensing mechanism of the DEF/c-MWCNTs/PPy sensor under tensile strain. (**a**–**c**) The SEM images of polyester fiber (warp yarn)-loaded c-MWCNTs and PPy with 0, 10 and 30% strain of warp sensor. (**d**–**f**) The SEM images of cotton fiber (warp yarn)-loaded c-MWCNTs and PPy with 0, 10 and 30% strain of weft sensor. (**g**–**i**) The distribution of the c-MWCNTs and PPy with 0, 10 and 30% strain of warp sensor. (**j**–**l**) The distribution of the c-MWCNTs and PPy with 0, 10 and 30% strain of weft sensor.

To further explore the working mechanism of the DEF/c-MWCNTs/PPy strain sensors, the morphological changes of strain sensors before and after repeated stretching/releasing tests were tracked using SEM (Figure 7). The original morphology of polyester fiber and cotton fibers are shown in Figure 7a,d, and it was observed that the c-MWCNTs and PPy formed a uniform aggregation on the surface of the fibers. However, when being stretched up to 0–10%, the PPy particles suffered tensile force, microcracks emerged and propagated on the surface of the fibers (Figure 7b,e), and larger stretching led to longer microcracks. If the strain between PPy exceeded the maximum allowable strain, the cohesive force between PPy disappeared and breakage between the PPy occurred (Figure 7c,f) [46]. In this situation, the c-MWCNTs will replace PPy to play a major role in sensing and connecting the conductive path. Under further stretching, the c-MWCNTs were stretched, straightened, and slid, until the breakage between c-MWCNTs happened. A possible mechanism for DEF/c-MWCNTs/PPy strain sensors was presented in Figure 7g–i (warp sensor) and Figure 7j–l (weft sensor), the conductive network of PPy was damaged when stretching to 10%, thus the sensors loaded with PPy are suitable for monitoring small strains (0–10%). However, the c-MWCNTs are flexible and stretchable, and the sensors fabricated by c-MWCNTs are fit to detect large strain (10–80%).

### 3.4. Applications of DEF/c-MWCNTs/PPy Strain Sensor for Human Motion Monitoring

**Figure 8:**
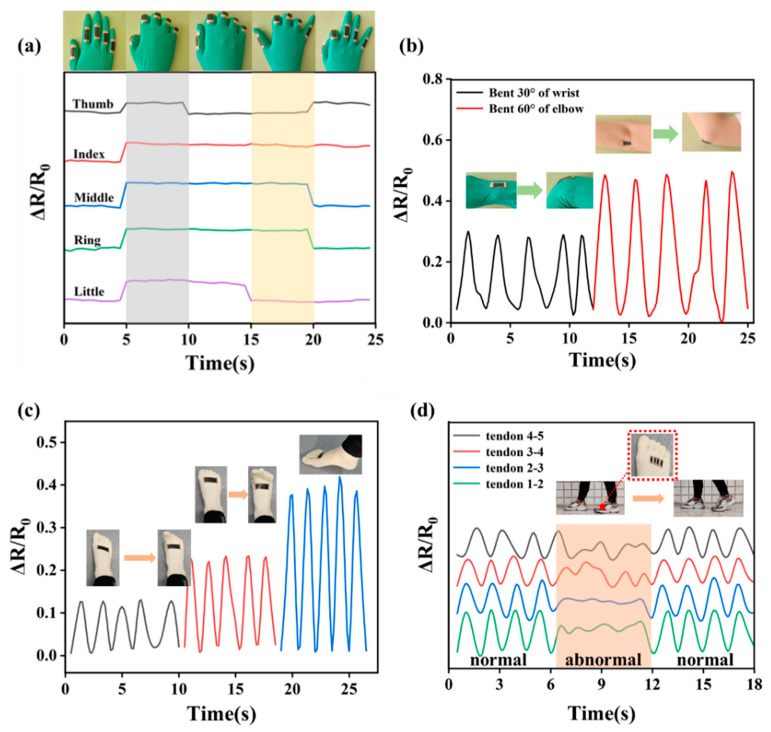
Applications of DEF/c-MWCNTs/PPy sensor in monitoring the joints motions: the RRV signal of warp strain sensor for monitoring (**a**) fingers joints motions and (**b**) wrist and elbow joints motions. The RRV signal of weft strain sensor for monitoring (**c**) toes motions and (**d**) walking.

**Figure 9:**
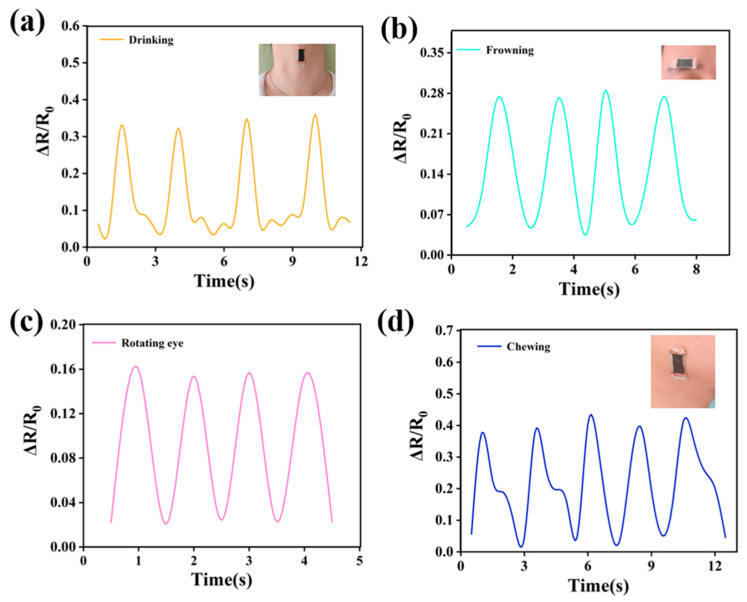
Applications of DEF/c-MWCNTs/PPy sensor in monitoring various subtle human motion: the RRV signal of DEF/c-MWCNTs/PPy warp sensor in monitoring (**a**) drinking and (**b**) frowning. The RRV signal of DEF/c-MWCNTs/PPy weft sensor in monitoring (**c**) eye movements and (**d**) facial muscle movements.

Owing to the excellent electromechanical properties and good response to stretching of sensors demonstrated by the above test, DEF/c-MWCNTs/PPy strain sensors were a promising candidate for use in wearable devices. Therefore, various human motions employing different body parts including large motions and subtle motions were detected by sensors made of DEF/c-MWCNTs/PPy (Figure 8 and Figure 9). Figure 8a displayed the RRV signal of warp sensor (3 cm × 1 cm) used for simultaneously monitoring five finger motions. It is vividly depicted that the RRV curves were initially flat when fingers in a non-bending state, then when the five fingers were bent, the RRV signal increased correspondingly. Subsequently, the thumb was straightened and another four fingers remained in a bending state, the RRV curve of thumb decreased gradually to initial value and other curves kept stable except a slight changes in RRV signal arising from the fingers trembling. The signal exhibited a step-by-step process and could accurately match the simultaneous motion of five fingers, demonstrating a promising prospect in real-time motion monitoring. Figure 8b showed the ability of warp strain sensors in monitoring of wrist and elbow joints motions. The obtained RRV curve was stable, repeatable, and changed accordingly when the tester periodically bent/unbent his wrist or elbow in a specific angle.

Similarly, the weft sensor (6 cm × 2 cm) was attached to the tester’s instep to record RRV signal of the toes’ motions. As shown in Figure 8c, the acquired curve was precise and repeatable, which was consistent with the toes’ behaviors and can distinguish different toe motions. When the toes did different movements, the RRV signals of the sensor were significantly changed. The large-scale motion deformation corresponds to the large tensile strain of the sensor, resulting in an increased intensity of RRV value. Furthermore, another human motion, walking, was monitored by the weft strain sensor. Four weft sensors (2 cm × 0.5 cm) was attached to the tendons between the five toe joints (Figure 8d), and numbered 1–2, 2–3, 3–4, 4–5 from left to right, the insets showed the location of the sensors and the movements of tester during walking. It could be seen that the sensors presented stable, repeatable, and regular RRV signals during the continuously walking process. However, when the paces of the tester were messy and abnormal, the RRV curves were disorderly and no regularity followed. In consideration of the highly responsive property of DEF/c-MWCNTs/PPy strain sensors to human motion, it is indicated that the sensors could be used as an electronic device to monitor patients with motor dysfunction and Parkinson’s disease.

In addition, the subtle human motions such as drinking, chewing, frowning, and eye movements can also be promptly and accurately monitored by DEF/c-MWCNTs/PPy strain sensors (Figure 9). Figure 9a,b displayed the real-time resistance change of the warp sensor when a tester was drinking water or frowning, inset showed the sensors mounted around the throat or brow, which could detect the resistance changes obviously. The resistance signals were completely synchronized and regular when a person drinking water or frowning did so repeatedly. Figure 9c,d presented the DEF/c-MWCNTs/PPy weft sensors used for the eye and facial muscle motion detection, the resistance signals were rapidly responsive to the subtle motions of eyes rotating and chewing, which were totally repeatable.

**Figure 10:**
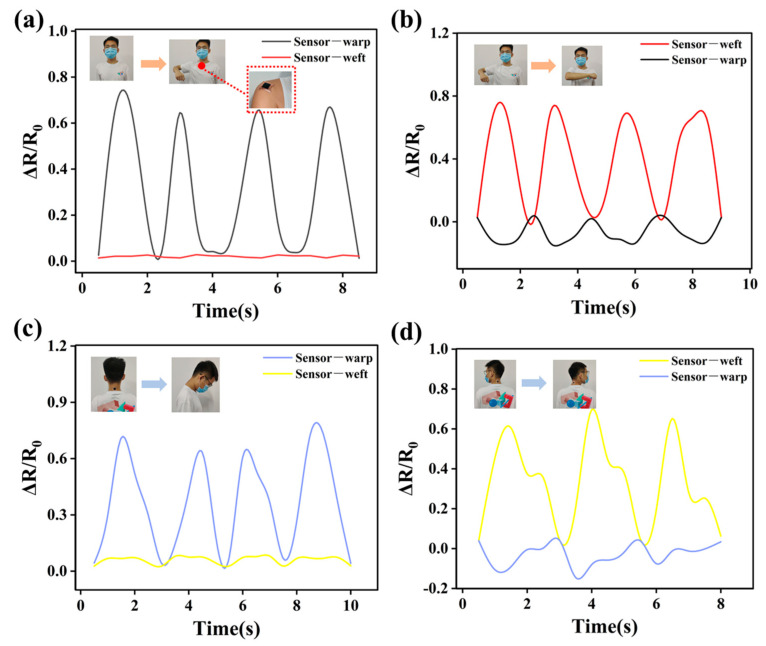
Applications of DEF/c-MWCNTs/PPy sensor in monitoring complicated human motions: the RRV signal of DEF/c-MWCNTs/PPy sensor in monitoring shoulder joint (**a**) up and down motions and (**b**) forth and back motions. The RRV singles of DEF/c-MWCNTs/PPy sensor in monitoring neck (**c**) up and down motions and (**d**) left and right motions.

As we already know, the body motions of shoulder joint and neck were multidimensional and complex, thus the conventional uniaxial strain sensor could not monitor those multiple-axis motions accurately. However, as a biaxial strain sensor, DEF/c-MWCNTs/PPy strain sensors possessed the unique anisotropic electro-mechanical ability of detect the resistance change of the warp and weft axes independently, which can distinguish various complex joint motions of the human body. To demonstrate the potential use of the DEF/c-MWCNTs/PPy sensor, complicated strain conditions in the human shoulder joint and neck and were detected. As presented in Figure 10a,b, the strain sensors (2 cm × 2 cm) were attached on the shoulder with the warp sensor along the direction of arm to detect the ΔR/R_0_ curves of shoulder joint under periodical up-down and forth-back motions. For the shoulder motions in the up-down direction, as expected, the RRV signal responded rapidly and repeatedly, and the sensing branch on the warp direction showed higher sensitivity (i.e., larger ΔR/R_0_), while the signal in the weft direction remained almost unchanged (Figure 10a), indicating a lager deformation in the warp-direction and a negligible change in weft strain. Not surprisingly, when the rotational movements were made of the repeated forth-back motions of the shoulder, a distinguished set of responses was recorded. It was noted that the ΔR/R_0_ curve in the weft direction regularly moved up and down, but the warp sensor exhibited a distinct negative resistance change, reflecting a large increase in tension at weft direction and a large compressive strain in the warp direction.

In terms of the relationship between the stretching direction and the direction of fixed sensor, four different RRV signal were obtained during bending-stretching motions of the neck, which was similar with the above motions of the shoulder. As shown in Figure 10c,d, the DEF/c-MWCNTs/PPy sensor mounted on the neck can successfully distinguish two unique neck motions and the multidimensional sensor was also shown to be highly responsive to sequential repeated up-down and left-right motions of the neck in both the weft and warp directions. When up and down motions of neck were conducted, the RRV curve of warp direction repeatedly moved up and down, while that in the weft direction kept almost flat. Upon repeated swinging left-right of the neck, ΔR/R_0_ in the weft direction showed an up and down fluctuations in the positive side, while warp sensor presented a small negative response. These results suggested that this real time anisotropic response to human body motion in different directions gave the DEF/c-MWCNTs/PPy strain sensors the unique ability as a wearable sensing device to monitor complicated human body motions in multiple dimensions.

## 4. Conclusions

In summary, a novel multidirectional wearable strain sensor was successfully fabricated by integrating c-MWCNTs and PPy into a double elastic fabric through facile, scalable soaking and adsorption-oxidizing methods to achieve excellent monitoring of human body motions. The DEF/c-MWCNTs/PPy strain sensor displayed good electrical conductivity, relatively high sensitivity (the warp maximum GF of 5.2 and the weft maximum GF of 4.6, large workable strain range up to 80%), fast response (<100 ms), low creep, good cycling repeatability, and durability (over 800 stretching-releasing cycles). On the one hand, the DEF/c-MWCNTs/PPy strain sensor could be used for accurately detecting both subtle motions and dynamic large-scale motions, such as joint movement, walking, drinking and eye movements. On the other hand, the DEF/c-MWCNTs/PPy strain sensor possessed anisotropic electromechanical behaviors that can distinguish various complicated joint motions of the human body, such as shoulder joint and neck motions by monitoring body movements in both of warp and weft directions. Consequently, the DEF/c-MWCNTs/PPy strain sensor may open new potentials in the field of wearable electronic devices and real-time healthcare monitoring.

## Supplementary Materials

The following are available online at https://www.mdpi.com/article/10.3390/nano11092333/s1, Figure S1: SEM images of (**a**) cotton fibers loaded c-MWCNTs, (**b**) polyester fibers loaded c-MWCNTs, (**c**) cotton fibers loaded c-MWCNTs and PPy and (**d**) polyester fibers loaded c-MWCNTs and PPy, Figure S2: The Plots of RRV versus strain for DEF/PPy sensor, Figure S3: Strain-stress curves of pristine DEF: (**a**) The tensile tests machine, (**b**) The tensile curve of the warp direction and (**c**) The tensile curve of the weft direction, Table S1: Comparison of the key performance indicators for different dual axis strain sensors.
