# Supplementary material for: Highly Sensitive and Stretchable c-MWCNTs/PPy Embedded Multidirectional Strain Sensor Based on Double Elastic Fabric for Human Motion Detection
Source: Nanomaterials (Basel). 2021 Sep 8;11(9):2333. doi: 10.3390/nano11092333 (PMC8467426; doi:10.3390/nano11092333)
Supplement: Supplementary file 1 [file nanomaterials-11-02333-s001.zip › nanomaterials-1345368-SI-conversion (new).pdf]

nanomaterials

SUPPORTING INFORMATION

Highly Sensitive and Stretchable c-MWCNTs/PPy Embedded Multidirectional Strain Sensor Based on Double Elastic Fabric for Human Motion Detection

Huiying Shen [1], Huizhen Ke [2], Jingdong Feng [1], Chenyu Jiang [3], Qufu Wei [1] and Qingqing Wang [1,*]

[1] Key Laboratory of Eco-textiles, Ministry of Education, Jiangnan University, Wuxi 214122, China; shenhuiying_vicky@163.com (H.S.); 13101972828@163.com (J.F.); qfwei@jiangnan.edu.cn (Q.W.)
[2] Key Laboratory of Novel Functional Textile Fibers and Materials, Minjiang University, Fuzhou 350108, China; kehuizhen2013@163.com
[3] Department of Chemistry, North Carolina State University, Raleigh, NC 27695, USA; cjiang13@ncsu.edu
* Correspondence: qqwang@jiangnan.edu.cn; Tel.: +86-150-5227-5367

Table of Contents

Figure S1. SEM images of (a) cotton fibers loaded c-MWCNTs, (b) polyester fibers loaded c-MWCNTs, (c) cotton fibers loaded c-MWCNTs and PPy and (d) polyester fibers loaded c-MWCNTs and PPy.

Figure S2. The Plots of RRV versus strain for DEF/PPy sensor.

Figure S3. Strain-stress curves of pristine DEF: (a) The tensile tests machine, (b) The tensile curve of the warp direction and (c) The tensile curve of the weft direction.

Table S1. Comparison of the key performance indicators for different dual axis strain sensors.

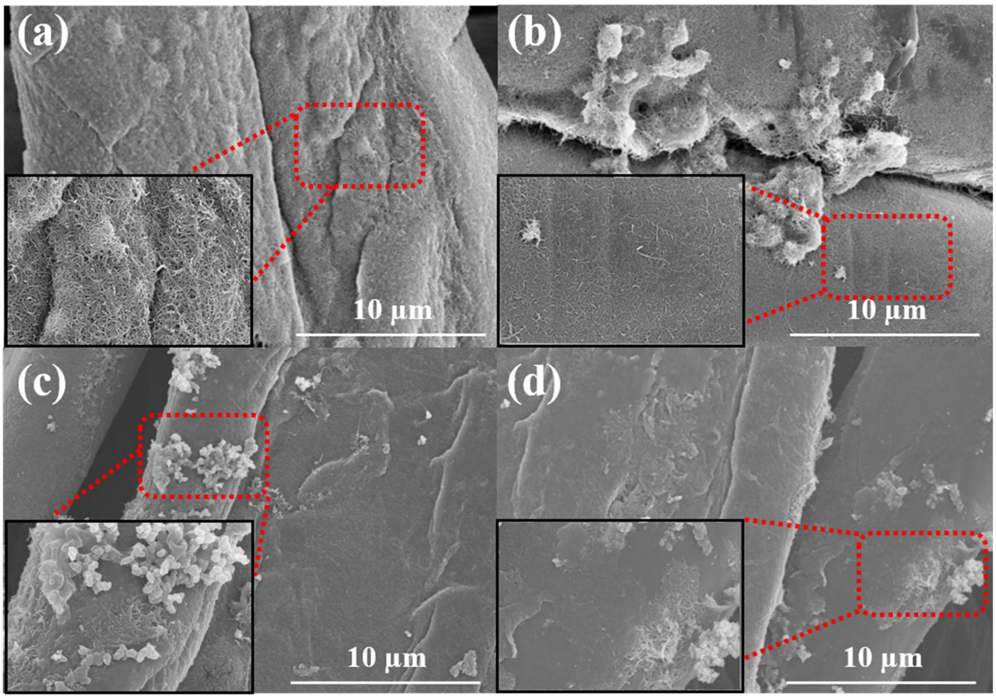

Figure S1. SEM images of (**a**) cotton fibers loaded c-MWCNTs, (**b**) polyester fibers loaded c-MWCNTs, (**c**) cotton fibers loaded c-MWCNTs and PPy and (**d**) polyester fibers loaded c-MWCNTs and PPy.

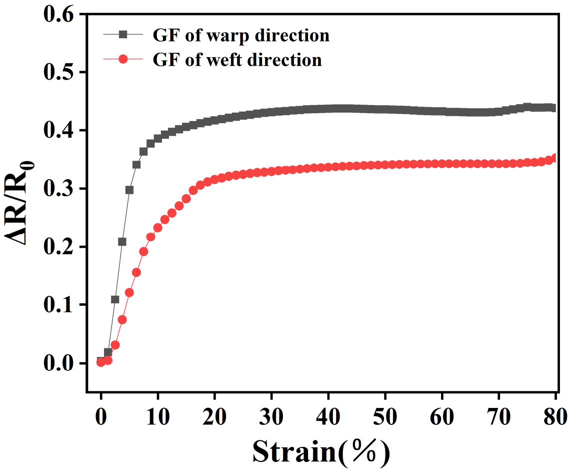

Figure S2. The Plots of RRV versus strain for DEF/PPy sensor.

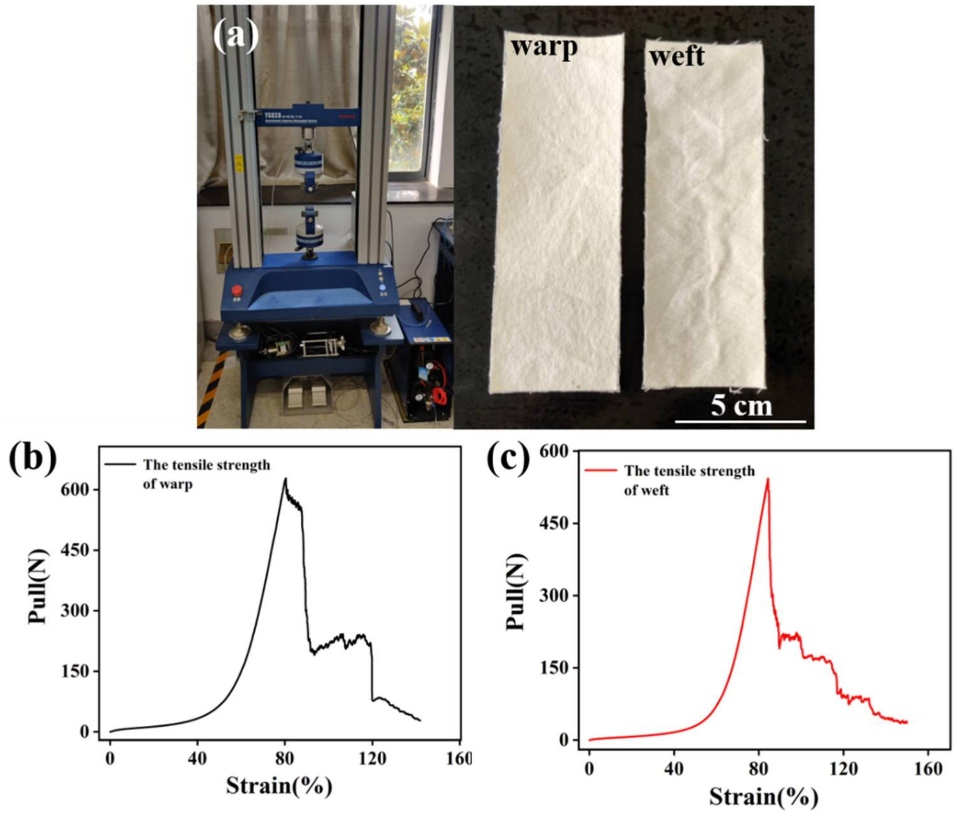

Figure S3. Strain-stress curves of pristine DEF: (**a**) The tensile tests machine, (**b**) The tensile curve of the warp direction and (**c**) The tensile curve of the weft direction.

Table S1. Comparison of the key performance indicators for different dual axis strain sensors.

Materials	Maximum strain (%)	GF	Ref.
stiffness-variant stretchable substrate/AgNW	60	21.1	[1]
Aligned cellulose fibers/PDMS	5	3.69–10.1	[2]
St-CCT/silicone	42	9.69–19.56	[3]
anisotropic carbon nanofiber films	30	180	[4]
Anisotropic CellF–CNT/PDMS	9	1.19	[5]
AgNWs fiber electrodes/polymer matrix	30	3.2	[6]
DEF/c-MWCNTs/Ppy	80	5.2	This work

[1] Ha, S.; Ha, S.; Jeon, M.B.; Cho, J.H.; Kim, J.M., Highly sensitive and selective multidimensional resistive strain sensors based on a stiffness-variant stretchable substrate. *Nanoscale* 2018, 10, 5105-5113.
[2] Chen, S.; Song, Y.; Ding, D.; Ling, Z.; Xu, F. Flexible and Anisotropic Strain Sensor Based on Carbonized Crepe Paper with Aligned Cellulose Fibers. *Adv. Funct. Mater.* 2018, 28, 1802547.
[3] Huang, P.; Yu X.; Li, Y.; Fu, Y.; Gan, D.; Taha, T.; Fu, Y.; Hu, N.; Fu, S. Architectural design of flexible anisotropic piezoresistive composite for multiple-loading recognization. *Compos. B. Eng.* 2020, 182, 107631.
[4] Lee, J.H.; Kim, J.; Liu, D.; Guo, F.; Shen, X.; Zheng, Q.; Jeon, S.; Kim, J.K., Highly Aligned, Anisotropic Carbon Nanofiber Films for Multidirectional Strain Sensors with Exceptional Selectivity. *Adv. Funct. Mater.* 2019, 29, 1901623.
[5] Wang, C.; Pan, Z.; Lv, W.; Liu, B.; Wei, J.; Lv, X.; Luo, Y.; Nishihara, H.; Yang. Q. A Directional Strain Sensor Based on Anisotropic Microhoneycomb Cellulose Nanofiber-Carbon Nanotube Hybrid Aerogels Prepared by Unidirectional Freeze Drying. *Small* 2019, 1805363.
[6] Cheng, Y.; Wang, R.; Zhai, H.; Sun, J. Stretchable electronic skin based on silver nanowire composite fiber electrodes for sensing pressure, proximity, and multidirectional strain. *Nanoscale* 2017, 9, 3834-3842.
